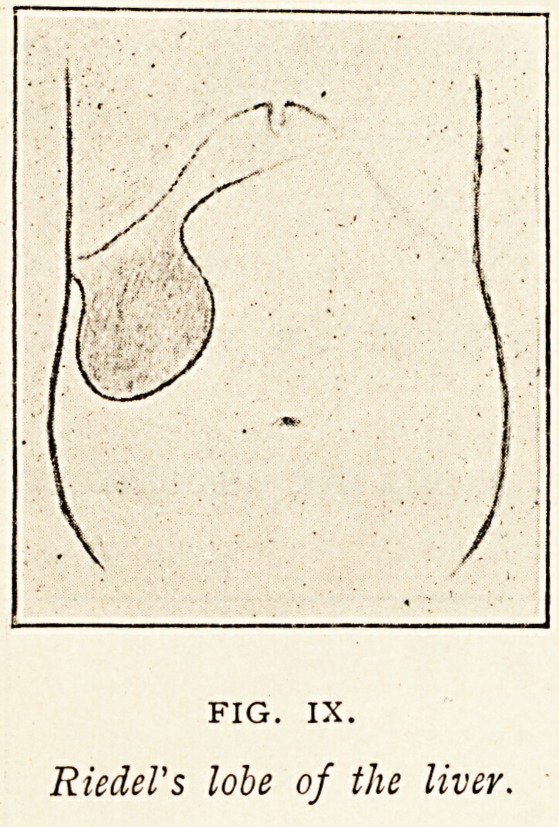# Some Cases of Displacement of Abdominal Viscera

**Published:** 1901-09

**Authors:** Theodore Fisher

**Affiliations:** Physician to Out-Patients to Bristol Royal Hospital for Sick Children; Pathologist to the Bristol Royal Infirmary.


### SOME CASES OF DISPLACEMENT OF ABDOMINAL VISCERA.

BY

Theodore Fisher, M.D., M.R.C.P.,

*Physician to Out-patients to Bristol Royal Hospital for Sick Children; Pathologist to the Bristol Royal Infirmary.*

Read before the Bristol Medico-Chirurgical Society, March 13th, 1901.

---

The accompanying illustrations, with one exception, are reproductions of rough sketches jotted down as memoranda of displacements of abdominal organs seen in the *post-mortem* room. Although the conditions represented may not, in the majority of instances, be of great practical importance, some at least possess a certain measure of interest.

Figure I. represents a diaphragmatic hernia in a female infant, aged six months, which was admitted into the Bristol Royal Infirmary in 1898, under the care of Dr. Waldo, for marasmus, and died soon after admission. The child was very emaciated, and weighed, at the time of the autopsy, only eight pounds. The whole of the left side of the chest was filled with intestines, which had found their way into the thorax by a deficiency in the posterior attachment of the diaphragm. An aperture which admitted two fingers existed where the diaphragm should have joined the external arched ligament. All the intestines, except the duodenum and the lower half of the colon and rectum, had passed into the chest. The left lung was completely collapsed, and the heart was displaced to the right of the sternum. The intestine remaining in the abdomen stretched from the aperture in the diaphragm to the anus in almost a straight line. The stomach was greatly dilated and reached to the pubes.[1]

FIG. I.

*Diaphragmatic Hernia. Nearly all the intestines are in the left pleural cavity.*

Diaphragmatic herniæ are most commonly congenital; and although the deficiency which allows the escape of the stomach or intestines from the abdominal cavity varies in size, it is most commonly small and situated posteriorly—as in the above case—at the position of the attachment of the diaphragm to the external arched ligament. When talking to Prof. Fawcett about the position of this deficiency, he mentioned to me that during the development of the diaphragm an aperture becomes formed at that point which is comparatively late in closing, and in some of the lower vertebrates, such as the reptilia and amphibia, it persists. Although, no doubt, the existence of this aperture in the human subject is as common on the right as on the left side, herniæ are much more common on the left owing to the fact that hollow viscera are in contact with the left half of the diaphragm. When an aperture is present it does not necessarily follow, however, that a hernia will occur. At least,

[1] It is to be regretted that the above diagram was not from a sketch, and therefore possibly does not accurately represent the position of the stomach.

the fact that patients may live until past middle-life before the prolapse occurs would lead one to think so.[1] Generally the most prominent symptoms are those of intestinal obstruction; but occasionally collapse associated with dyspnœa, and the discovery of a tympanitic note on the left side of the chest, may lead to the diagnosis of pneumothorax. More than once the stomach has been aspirated through the chest-wall.[2]

Figure II. also represents a congenital condition. The cæcum was attached to the lumbar vertebræ immediately below the duodenum. Posteriorly, the cæcum was devoid of peritoneum. The vermiform appendix, directed upwards and to the right, reached the margin of the ribs. The condition was obviously congenital and not produced by adhesions of an abnormally movable cæcum, but how the cæcum came to occupy a position over the lumbar vertebræ is not obvious. During development the cæcum moves from the left to the right hypochondrium, and from there down to the iliac fossa. It may become arrested in almost any part of its course, but mere arrest will not explain the position in this case. The cæcum lay outside what we may call its normal path. The practical interest of the case lies in the situation of the vermiform appendix. Inflammation of an appendix in that situation would lead to some difficulty of diagnosis. The displacement occurred in a man, aged 38 years, who died of septicæmia following cellulitis of the knee.

FIG. II.

*The cæcum is attached in front of the lumbar vertebræ, and the vermiform appendix is directed upwards to the right costal margin.*

[1] *Vide* case in a man aged 49, recorded by Goodhart, *Lancet*, 1893, i. 362.

[2] *Vide* case recorded by Hollis, *Lancet*, 1895, i. 1095.

Figure III. is a diagram illustrating a displacement of the cæcum of different character, but also dependent upon the persistence of a fœtal condition. The cæcum was displaced upwards, and lay partly on the anterior surface of a slightly enlarged liver. It was, however, not fixed in that position. The cæcum and ascending colon possessed a mesentery of sufficient length to allow the cæcum to be easily placed in the left iliac fossa. At the time of the autopsy the cæcum was situated—as shown in the diagram—in the right hypochondrium, but probably often occupied other positions. The long meso-cæcum present in this case is a persistent fœtal condition. During a great part of fœtal life the large intestine possesses a long mesentery. When the colon assumes the position it is to permanently occupy the mesentery becomes shortened, and commonly disappears from the ascending colon and cæcum. The displacement occurred in a woman, aged 32 years, who died of cardiac disease.

FIG. III.

*Freely movable cæcum, lying immediately below the right costal margin.*

Figure IV. represents the right half of the transverse colon and part of the stomach turned upwards, so as to overlie the anterior surface of the liver. The transverse meso-colon has been omitted for the sake of clearness. The cause of this upward displacement of the transverse colon and stomach was distension of the small intestine following carcinoma of the cæcum. Deep depressions in the liver substance marked the situations of the overlying colon and stomach, a fact that seemed to indicate that they had been for some little time in this abnormal position. I only remember having noticed a well-marked displacement of similar character on one other occasion; but possibly, if looked for, the condition might be met with not infrequently in minor degree. The other instance occurred in the *post-mortem* room only a few weeks before the case from which the above sketch was made. There the transverse colon only was turned upwards, but it lay on the liver on the left side of the falciform ligament as well as the right—that is to say, the left half of the transverse colon was situated where a portion of the stomach was in the above case.

FIG. IV.

*A portion of the transverse colon and of the stomach lying on the upper surface of the liver.*

Figure V. illustrates a condition of no very great interest, and it will be referred to only briefly. The transverse colon was adherent to the back of the uterus, and lay behind the small intestines. The transverse meso-colon was probably abnormally long, and the great omentum, involved in pelvic inflammation, had contracted until the transverse colon came into contact with the uterus.

FIG. V.

*Transverse colon adherent to the back of the uterus and lying behind the small intestine.*

The condition represented in Figure VI. is of entirely different character. The sketch roughly illustrates the position of some of the abdominal organs in an infant, aged 14 months, which died of tetany. Abnormal distension of the abdomen had been great, and had lasted for several weeks. The liver was so much displaced backwards and downwards that the posterior surfaces rested on the right iliac crest. The right kidney had been pushed before the liver into the right iliac fossa. The cause

FIG. VI.

*Liver displaced backwards, pushing the right kidney into the right iliac fossa.*

of this displacement was great distension of the stomach and intestines. This distension is, unfortunately, not shown in the diagram. The close connection of the liver with the right kidney no doubt plays an important part in the causation of movable kidney. Pressure exerted from outside the abdomen may apparently displace the liver directly downwards, and the kidney be carried downwards with it, or —as in this case—the liver may be rotated backwards and downwards by pressure brought to bear upon it from within the abdomen. Examination of the relation of the liver to the kidney would lead one to think that pressure from below upon the anterior half of the liver, producing rotation, would be much more likely to displace the kidney than external pressure. The most common effect of external pressure is to compress the liver at a level below the line of its contact with the kidney. External pressure must tend in many instances to tighten the grasp of the liver upon the kidney, rather than displace it. Possibly repeated flatulent distension of the abdomen may, when occurring in early life, be sufficient to loosen the normal attachments of the right kidney.

FIG. VII.

*Acute dilatation of the stomach.*

Figure VII. is an example of acute dilatation of the stomach. As shown in the diagram, the stomach reached the left iliac fossa, and extended also to the right of the middle line, overlapping and hiding from view all the intestines with the exception of the ascending colon. There was no obstruction of the pylorus, and the duodenum shared to some extent in the dilatation. The clinical history gave no indication of the time of onset of the dilatation. It occurred in a woman, aged 36, admitted, under the care of Mr. Munro Smith, in 1898, with swelling of the left eyelid. Cerebral symptoms set in, and the patient died. A suppurating thrombus was found in the left cavernous sinus, for which no cause could be found.

Figure VIII. represents an extreme degree of a not uncommon condition. The transverse colon is greatly distended as a consequence of malignant growth of the sigmoid flexure. Part of the distended colon, most of the stomach, and a small portion of the liver were the only viscera seen on the anterior aspect of the abdomen. The stomach, which was adherent to the transverse colon, had been drawn downwards, and its lowest point was situated slightly below and to the right of the umbilicus. The condition occurred in a woman aged 39 years.

FIG. VIII.

*Greatly dilated transverse colon in a case of stricture of the sigmoid flexure.*

Figure IX. is not a diagram of a displaced organ, but is reproduced here because it represents a condition which appears to be frequently mistaken for a displaced kidney. It is the so-called Riedel's lobe of the liver, an appendage situated generally over the gall-bladder or just outside it. The lobe is said to be associated with gallstones, and to be produced by concomitant dilatation of the gall-bladder. The gall-bladder as it dilates is thought to drag a tongue of liver substance downwards and forwards with it. Six years ago two examples of the lobe occurred in the *post-mortem* room of the Bristol Royal Infirmary within a few weeks of one another, but I did not make a sketch of either. The illustration represents what was felt during life in a man, aged 44, who was in the Bristol General Hospital in 1894, under the care of Dr. Harrison. Ascites, due probably to chronic peritonitis, had been present. The abdomen was an easy one to palpate, allowing the limits of the tumour and its connection with the liver could be clearly defined. It was looked upon as a congenital abnormality, and when the two other examples were met with in the *post-mortem* room, not being aware at the time of Riedel's description of this lobe, or of his explanation of its causation, I probably somewhat hastily concluded that they also were congenital. The presence or absence of gallstones is unfortunately not recorded. While, however, the association of gallstones which has been noticed with Riedel's lobe is interesting, and seems to be a reasonable explanation of the existence of the lobe, it is well to bear the fact in mind that the anterior border of the liver presents considerable variability in outline. For example, the degree to which the left lobe is cut off from the right, and its downward projection into the epigastrium, differs considerably in different cases. This variability in the shape of the liver suggests that a downward projection in the position of Riedel's lobe may be congenital, and the association noticed with gallstones merely a coincidence.[1]

FIG. IX.

*Riedel's lobe of the liver.*

[1] NOTE.—Since writing the above I have read an article by Glénard,[2] in which the causation of Riedel's lobe of the liver is briefly discussed. Glénard considers that a floating lobe is generally associated with (non-cirrhotic), enlargement of the liver and that it is produced by the pressure of the costal margin upon the upper surface of the liver, in cases where there has been tight-lacing of a corset or the pressure of a belt. The frequency with which pressure of this character produces a line of atrophy upon the upper surface of the liver is well known, and when the anterior margin of the liver happens to be abnormally curved, it is possible that pressure may partially detach a portion and produce a floating lobe.

[2] *Revue des maladies de la nutrition*, November, 1898.

## Figures and Tables

**FIG. I. f1:**
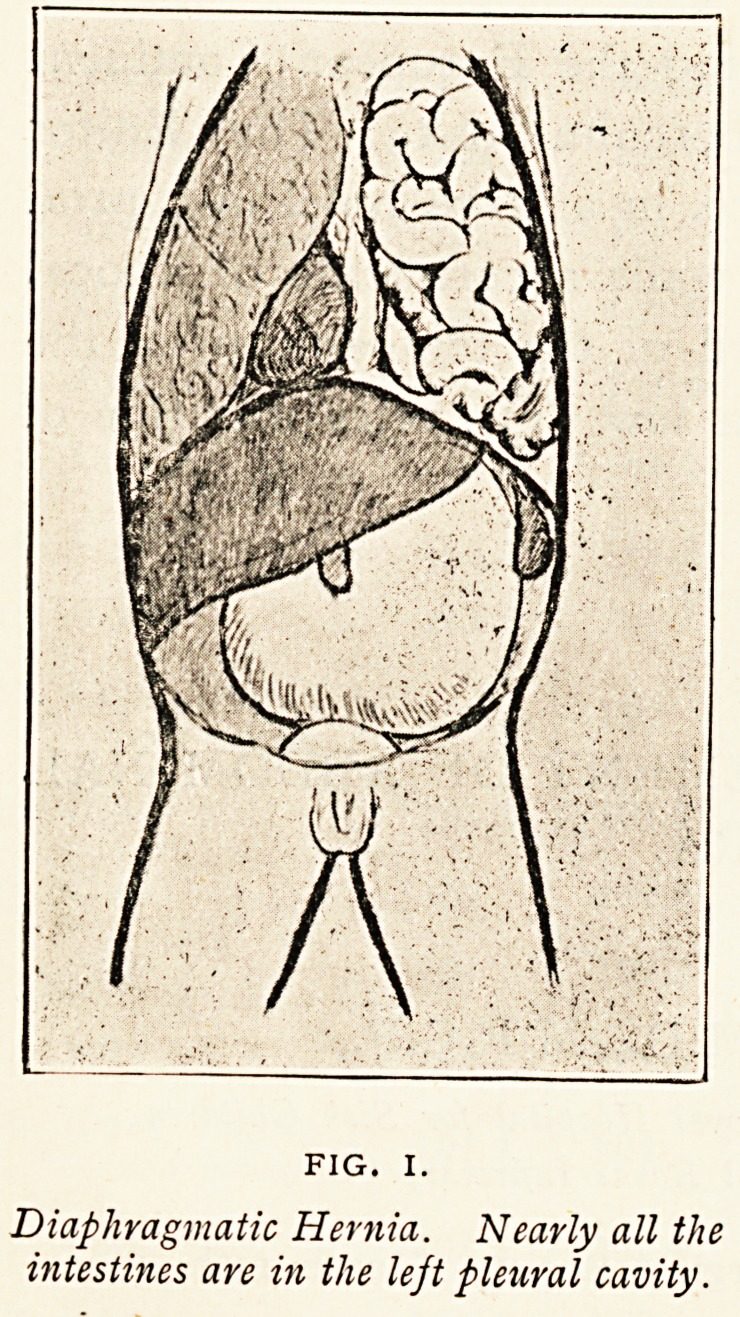


**FIG. II. f2:**
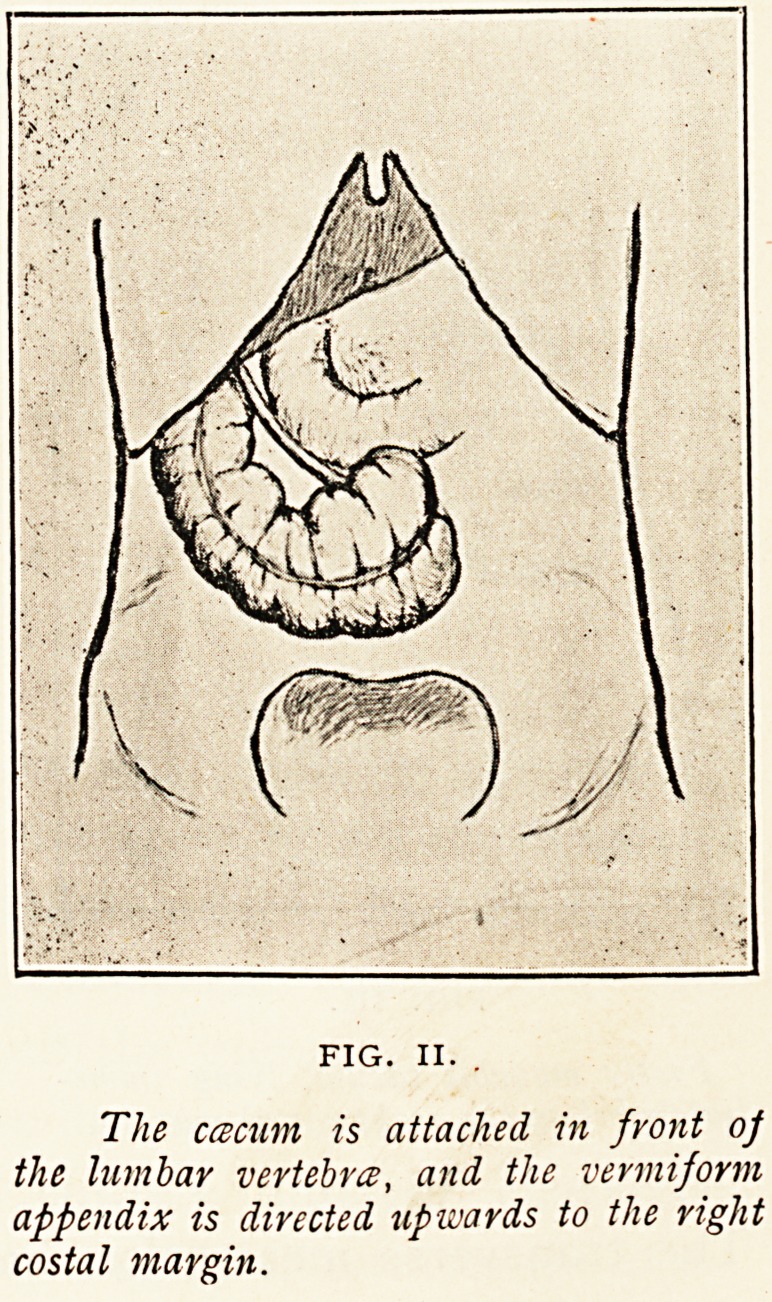


**FIG. III. f3:**
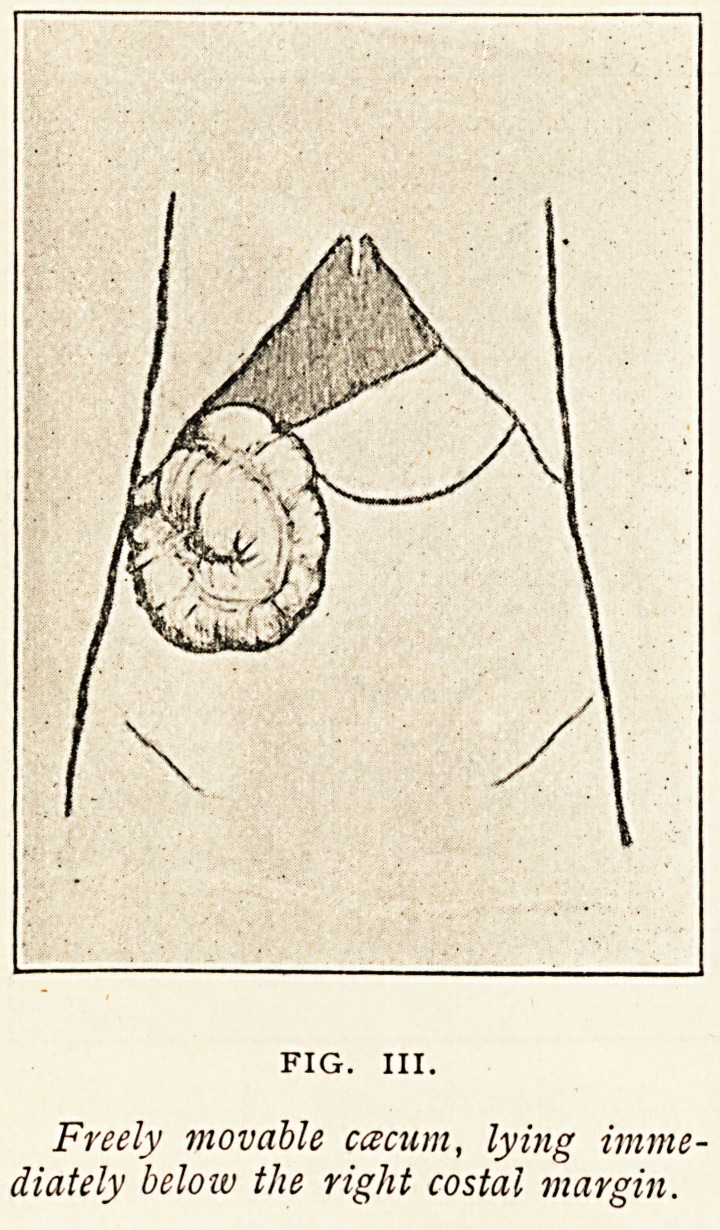


**FIG. IV. f4:**
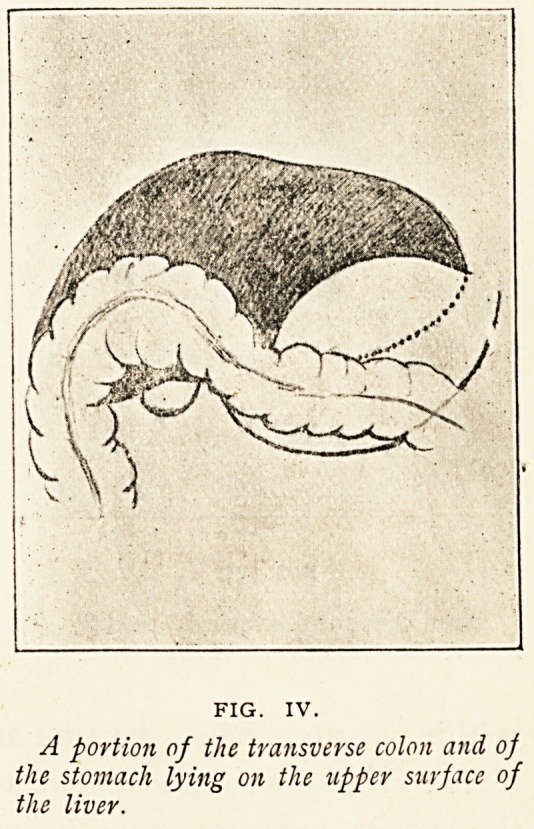


**FIG. V. f5:**
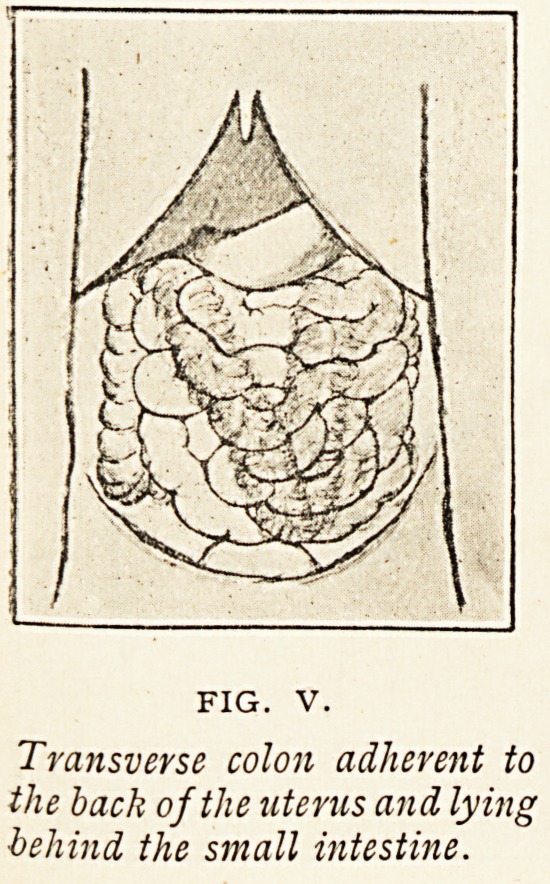


**FIG. VI. f6:**
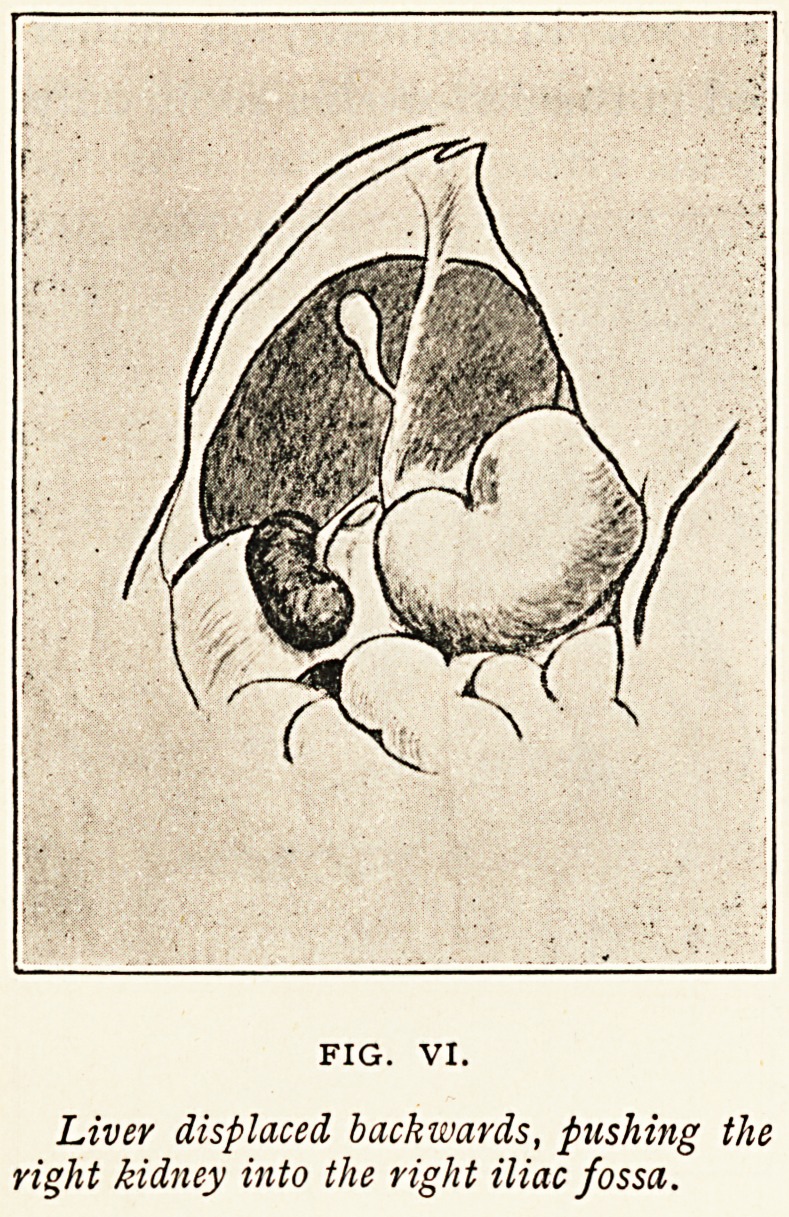


**FIG. VII. f7:**
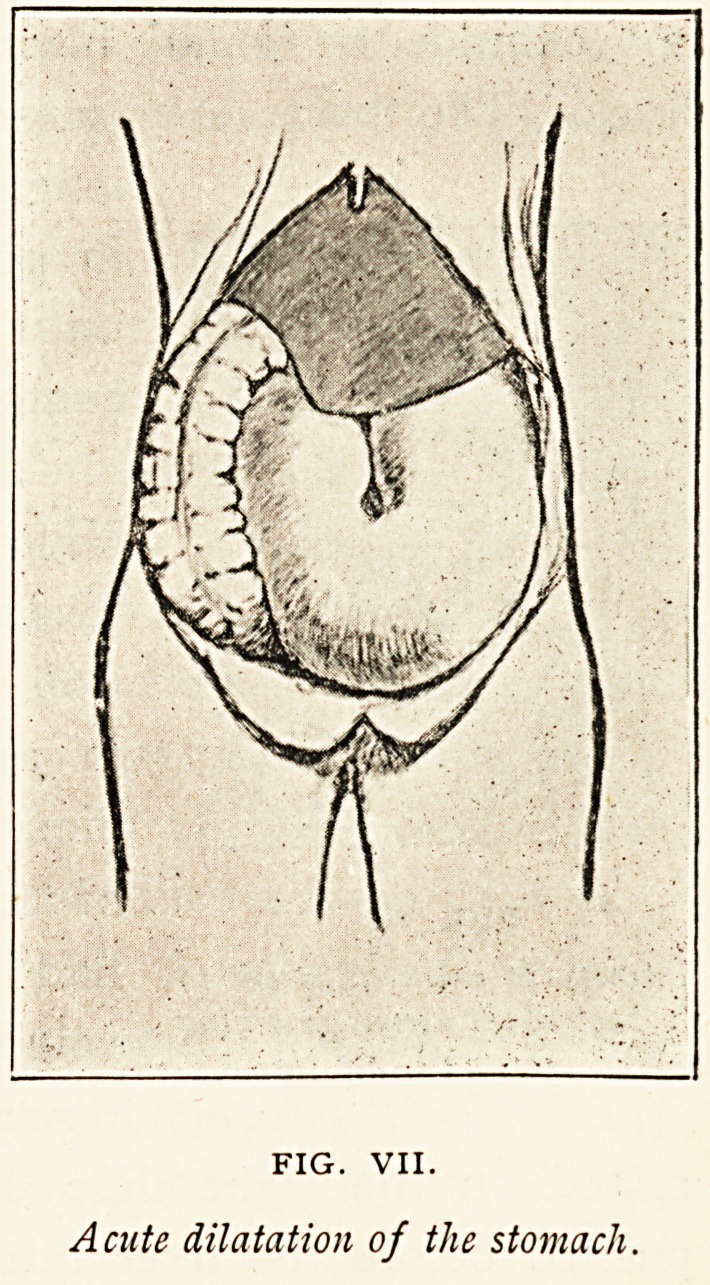


**FIG. VIII. f8:**
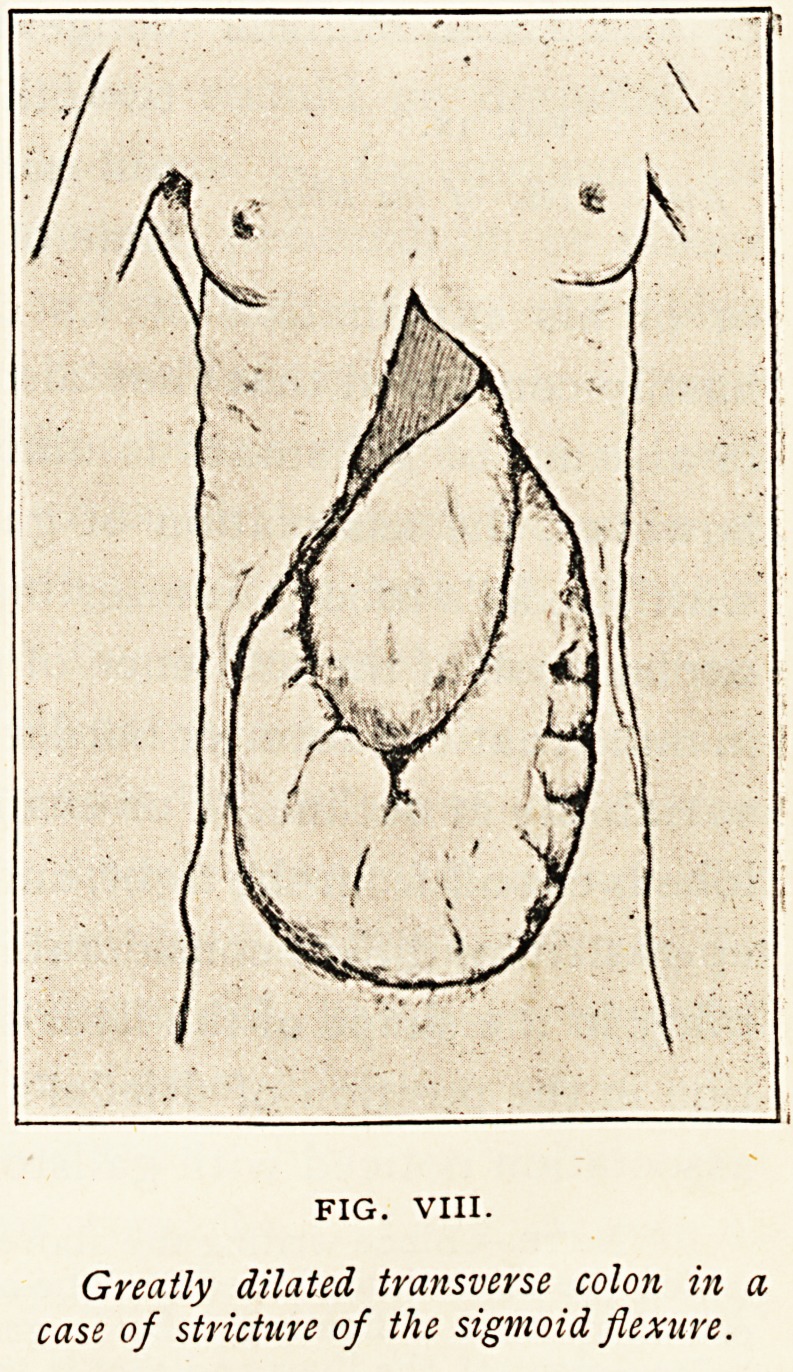


**FIG. IX. f9:**